# N-Type Nanosheet FETs without Ground Plane Region for Process Simplification

**DOI:** 10.3390/mi13030432

**Published:** 2022-03-11

**Authors:** Khwang-Sun Lee, Jun-Young Park

**Affiliations:** School of Electronics Engineering, Chungbuk National University, Cheongju 28644, Korea; ksunlee@chungbuk.ac.kr

**Keywords:** band-to-band tunneling, epitaxial growth, ground plane region, gate-all-around field-effect-transistors (GAA FETs), nanosheet FETs (NS FETs), parasitic channel leakage, punch-through

## Abstract

This paper proposes a simplified fabrication processing for nanosheet Field-Effect Transistors (FETs) part of beyond-3-nm node technology. Formation of the ground plane (GP) region can be replaced by an epitaxial grown doped ultra-thin (DUT) layer on the starting wafer prior to Si_x_/SiGe_1−x_ stack formation. The proposed process flow can be performed in-situ, and does not require changing chambers or a high temperature annealing process. In short, conventional processes such as ion implantation and subsequent thermal annealing, which have been utilized for the GP region, can be replaced without degrading device performance.

## 1. Introduction

To suppress short-channel effects (SCEs), which are an important concern with aggressive device scaling, semiconductor devices have evolved from 2-dimensional (2-D) structures to 3-dimensional (3-D) architectures. This has further benefits. For example, due to their superior gate controllability, FinFETs have lower subthreshold swing (*SS*) and off-state leakage (*I*_OFF_) than planar devices [1,2,3]. However, as device scaling approaches extreme levels, it is becoming difficult to control SCEs using FinFETs. As a solution, nanosheet FETs (NS FETs), which have multiple channels with a gate-all-around (GAA) backbone structure, have been introduced as beyond-FinFETs [4,5,6]. However, even though NS FETs have shown better suppression of SCEs than FinFETs, as well as better output performance, there are still many difficulties related to mass production. Producers such as Samsung Electronics Inc. and TSMC Inc. are planning the mass production of NS FETs in 2022 and 2023, respectively. However, it is unknown whether the yield will be sufficient. The reason for this lies in difficulties in the fabrication processing of the NS FETs. For example, surface roughness scattering stemming from germanium diffusion among the nanosheets, striction stemming from adhesion between nanosheets [6], residue formation (e.g., TiN or Si_3_N_4_) due to uncontrollable wet etching, unwanted void formation during metal gate filling [7], etc., are very challenging problems in current fabrication processing.

In addition, with respect to source/drain (S/D) modules, the contact depth as well as inner spacer thickness needs to be optimized [8,9]. It should be noted in particular that, unlike bulk FinFETs, which have already been mass produced, NS FETs have a parasitic channel underneath the first floor nanosheet [10,11,12,13]. Hence, even though NS FETs have multiple channels that are completely surrounded by metal gates (i.e., GAA), increased *I*_OFF_ stemming from the parasitic channel is inevitable. V. Jegadheesan et al., have suggested that applying a ground plane (GP) region can effectively minimize the *I*_OFF_ in the parasitic channel [14]. However, as we have mentioned above, the fabrication process flow of NS FETs is very sensitive. For example, ion implantation and rapid thermal annealing (RTA) for GP region formation are associated with an unwanted non-uniform doping profile [15,16]. In this context, it would be better, in terms of device variability and yield, if the conventional processes could be replaced with an alternative process. However, recently, research papers covering the fabrication process of NS FETs have been modest in number.

In this letter, we propose a fabrication process flow for NS FETs. The proposed process flow does not require ion implantation or additional thermal treatment, which are conventionally performed to form the GP region. Alternatively, a doped ultra-thin (DUT) layer is epitaxially grown on the starting wafer in-situ before Si_x_/SiGe_1−x_ stack formation. This achieves process simplification, improved variability, and improved yield during the fabrication of NS FETs.

## 2. Materials and Method

**Figure 1:**
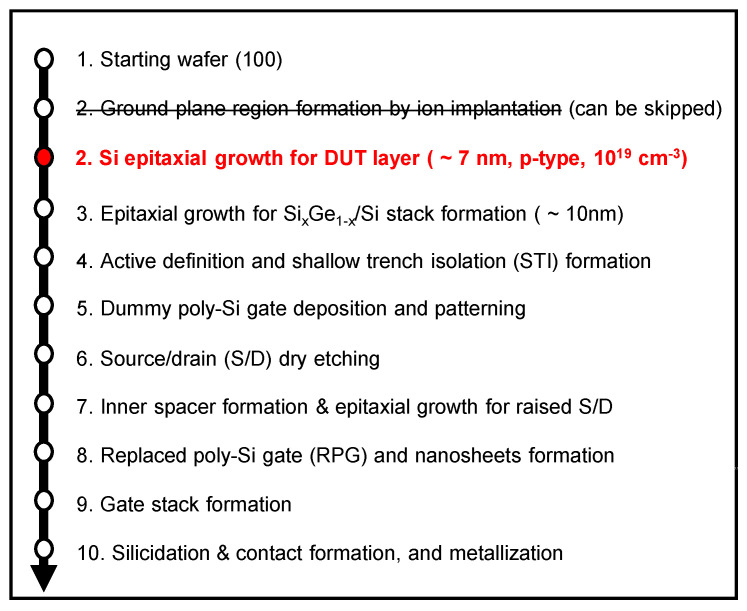
Fabrication process flow of proposed NS FETs including epitaxially grown DUT layer prior to formation of the Si_x_Ge_1−x_/Si stack. The bolded step two can be alternatively added instead of the under-lined ion implantation and annealing process.

The proposed fabrication process flow is summarized in Figure 1. In the conventional NS FETs fabrication process, ion implantation and thermal annealing are performed in dashed step two for GP region formation. However, the process can be replaced by Si epitaxial growth for DUT layer formation, as described in the bolded step two. The DUT layer is doped with a p–type dopant (i.e., boron) to minimize the parasitic channel and punch-through underneath the first floor nanosheet. The thickness of the DUT is similar to the thickness of silicon and Si_x_Ge_1−x_ layers, which are epitaxially grown as nanosheets and sacrificial layers, respectively. Hence, the formation of the DUT layer is not problematic under current processing technology. Above all, it should be noted that the processing from steps one to three in Figure 1 can be performed in-situ without changing chambers.

A 3-D simulator (Synopsys Sentaurus, Mountain View, CA, USA) was utilized to simulate fabrication processing and device characteristics. The drift-diffusion carrier transport equation was combined with the Poisson equation, and the density-gradient model was considered to reflect the quantum confinement effect of the nanosheet channels [17,18,19,20]. The Slotboom model was included for doping-dependent bandgap narrowing in the overall region [21]. Thin layer models such as inversion and accumulation layer mobility model (IALMob) were included to reflect impurity and phonon scattering [22]. In addition, the Shockley-Read-Hall (SRH) and non-local band-to-band tunneling (BTBT) recombination models were included to reflect gate-induced drain leakage (GIDL) during the simulations [22].

**Figure 2:**
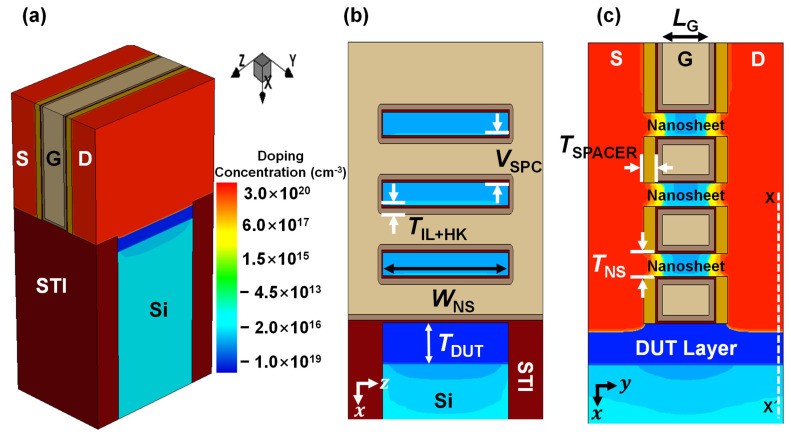
(**a**) Simulated NS FET device structure including the epitaxially–grown DUT layer on the starting wafer. Cross–sectional view of the proposed NS FET with a cut along the (**b**) gate and (**c**) channel directions, respectively.

Figure 2 shows the backbone structure of the NS FETs used in the process simulation. Channel thickness (*T*_CH_) and width (*W*_NS_) were 5 nm and 45 nm, respectively. To elaborate, the dielectric constant of the HfO_2_ gate dielectric and effective-oxide-thickness (EOT) were assumed to be 25 and 0.7 nm, respectively. In terms of doping concentration, epitaxially grown S/D regions were doped with arsenic at 3 × 10^20^ cm^−3^ [14,23]. The three nanosheet channels and silicon substrate (*N*_Sub_) were lightly doped with boron at 1 × 10^17^ cm^−3^ and 1 × 10^16^ cm^−3^, respectively. The doping concentration of the DUT layer was 1 × 10^19^ cm^−3^ of boron. The work-function of the titanium nitride for the metal gate was 4.5 eV. Detailed device parameters used for the simulations are summarized in Table 1.

**Figure 3:**
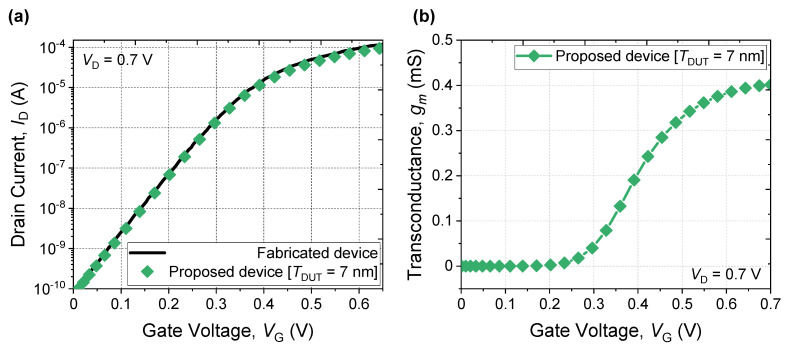
(**a**) Calibration of *I*_D_-*V*_G_ curve with the fabricated device in Reference [6]. (**b**) Transconductance of (**a**).

Then, the simulated *I*_D_-*V*_G_ was carefully calibrated based on fabricated devices (i.e., such as doping concentration, structure, and gate work function) reported in [6], as shown in Figure 3a. Threshold voltage (*V*_TH_) was extracted using a constant current method at *I*_D_ of 100 nA. Multiple *V*_TH_ characteristic can be visible when series resistance of NS FET is high due to defect existence or low doping concentration of S/D regions, as well as excessively long *V*_SPC_. However, there was no observable multiple value of *V*_TH_ observed in the subthreshold region. Hence such concerns were not problematic, as shown in Figure 3b.

## 3. Results and Discussion

**Figure 4:**
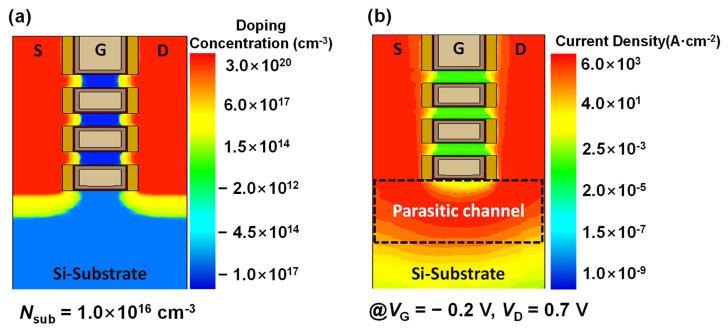
Simulated (**a**) doping profile distribution and (**b**) current density of a NS FET without a DUT layer during off–state.

Figure 4a shows the simulated doping profile during the off-state when the GP region as well as the DUT layer were not included. An unwanted parasitic channel and punch-through were formed underneath the first floor nanosheet. These concerns led to increased *I*_OFF_ during the off-state (Figure 4b).

**Figure 5:**
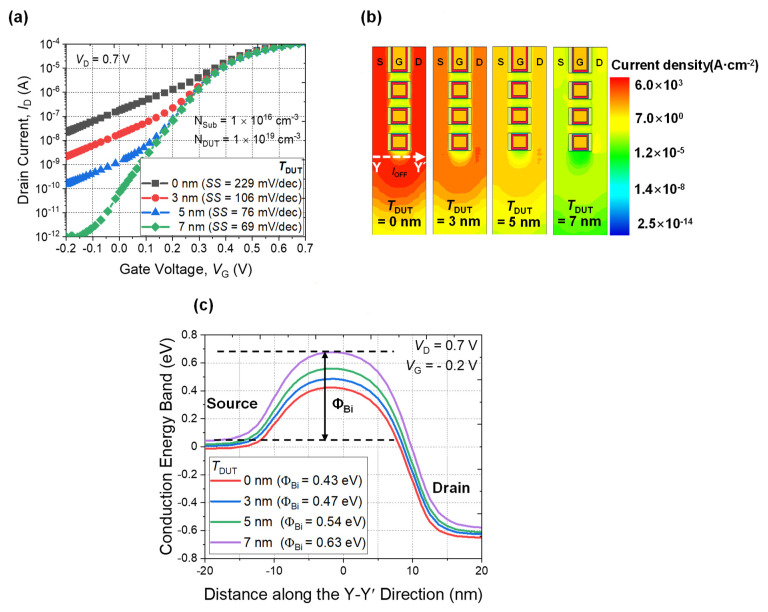
(**a**) Simulated *I*_D_-*V*_G_ characteristic of NS FETs with various thicknesses of DUT layers without GP implantation and subsequent annealing. (**b**) Simulated parasitic current distribution profiles with various thicknesses of DUT layers. (**c**) Energy band diagram of parasitic channel along the Y–Y′ direction in (**b**). Conduction energy band height increases as *T*_DUT_ increases.

Figure 5a shows the simulated transfer characteristics of the NS FETs with various thicknesses of DUT layers (*T*_DUT_) from 0 nm to 7 nm. As the thickness of the DUT layer increases, *I*_OFF,_ as well as *SS*, improve. The *I*_OFF_ extracted at *V*_G_ = −0.2 V was 21.8 nA with a 0 nm DUT layer but improved to 0.98 pA with a 7 nm DUT layer. Figure 5b shows the current density profile of the NS FETs in Figure 5a extracted at the off-state with *V*_D_ = 0.7 V, *V*_G_ = −0.2 V. As the *T*_DUT_ increases, *I*_OFF_ flowing through the parasitic channel (Y–Y′ direction) can be suppressed, aided by the increased energy barrier height (Φ_bi_ of the parasitic channel), as shown in Figure 5c. In addition, punch-through can be suppressed by applying a DUT layer. 

**Figure 6:**
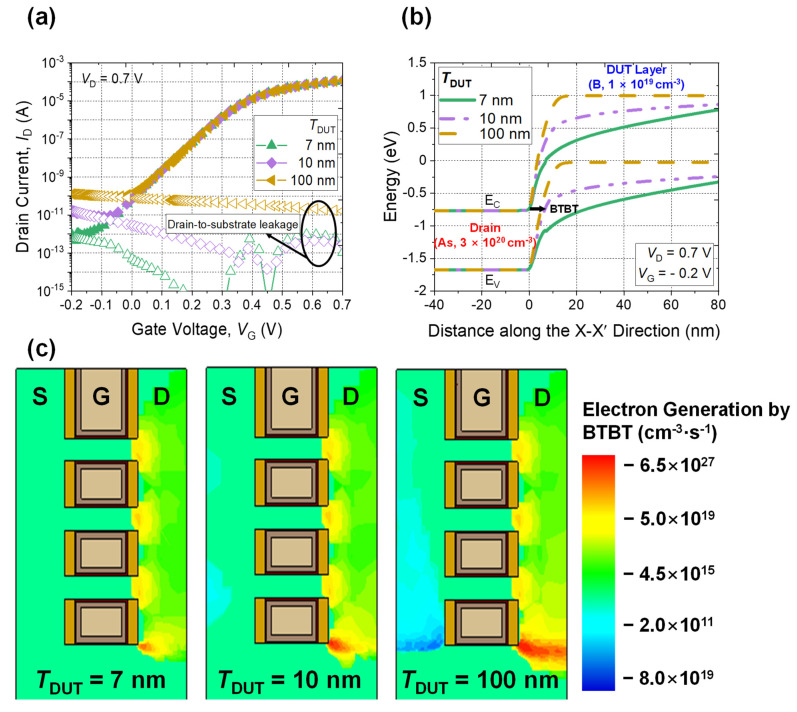
(**a**) Simulated *I*_D_-*V*_G_ characteristic and (**b**) extracted energy band diagram of NS FETs (X–X′ direction in Figure 2c) with various thicknesses of DUT layers greater than 7 nm. (**c**) Electron generation rates with various *T*_DUT_.

However, when *T*_DUT_ is thicker than 7 nm, the *I*_OFF_ increases (Figure 6a). Considering the *I*_OFF_ value in the range of *V*_G_ of –0.2 V to 0 V are identical to the drain–to–substrate leakage current, the source of the *I*_OFF_ increase is not the parasitic channel, but rather the BTBT between the drain and the DUT layer. In other words, when *T*_DUT_ is thicker than 7 nm, the DUT forms a p-n diode between the drain and the DUT layer, as shown in Figure 6b. During the off-state, the reverse biased p-n diode triggers BTBT of electrons, and increases the *I*_OFF_. Figure 6c shows the simulated rate of electron generation between the drain and the substrate by the BTBT. As the *T*_DUT_ increases, there is a noticeable increase in electron generation by the BTBT. As a result, it can be concluded that a 7 nm thickness DUT layer is optimal to avoid unwanted increasing *I*_OFF_.

**Figure 7:**
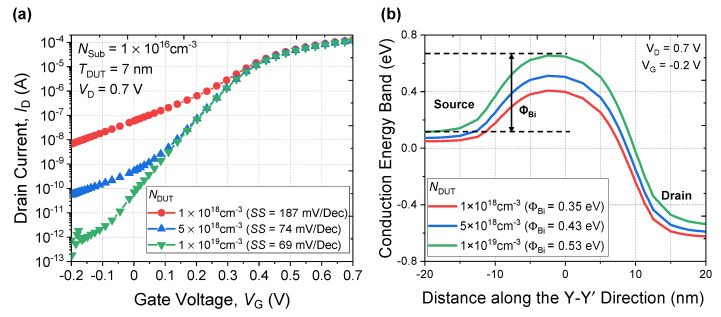
(**a**) *I*_D_-*V*_G_ characteristic with different *N*_DUT_ layer. (**b**) Energy band diagram of the parasitic channel.

Figure 7a shows a simulated *I*_D_-*V*_G_ curve for various doping concentrations (*N*_DUT_) of the DUT layer. When the *N*_DUT_ is a low doping concentration of 1 × 10^18^ cm^−3^, the *I*_OFF_ cannot be controlled due to increased *SS*. However, as the *N*_DUT_ increases, leakage current through the parasitic channel can be suppressed by the increased built-in potential (Φ_bi_) from 0.39 eV to 0.59 eV, as shown in Figure 7b. In addition, suppression of punch-through is possible with increased substrate doping concentration.

**Figure 8:**
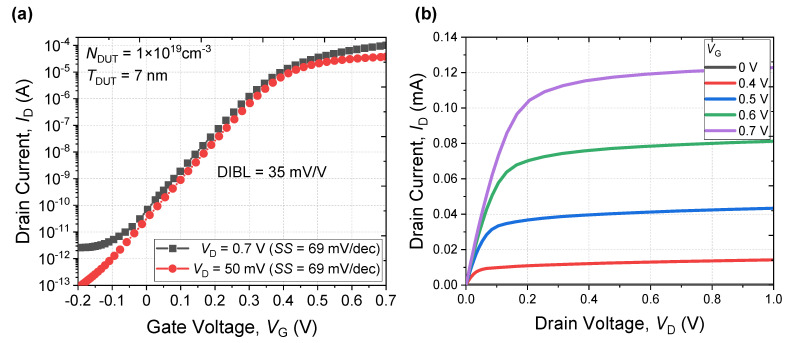
(**a**) Extracted *I*_D_-*V*_G_ and (**b**) *I*_D_–*V*_D_ characteristics of NS FET with DUT layer.

Figure 8 shows extracted *I*_D_-*V*_G_ curve at *V*_D_ = 50 mV and 0.7 V, as well as *I*_D_-*V*_D_ curve. The drain-induced barrier lowering (DIBL) of the proposed NS FET is 35 mV, which a low value compared with the value of 33 nm of the planar FET [19] or 15 nm of FinFET [24], as summarized in Table 2. In other words, the superior gate controllability of the NS FET does not degrade even when a DUT layer is applied. 

**Figure 9:**
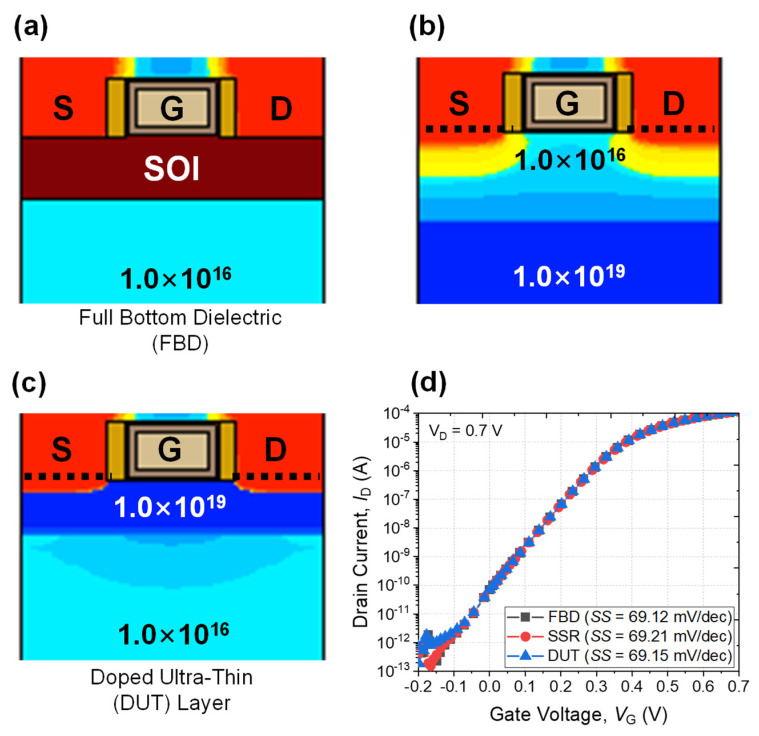
Various device structures without a GP region. NS FET with (**a**) full bottom dielectric (FBD) [25], (**b**) super steep-retrograde (SSR) region [14], and (**c**) the proposed DUT layer. (**d**) Simulated *I*_D_-*V*_G_ characteristics of the respective device structures.

Figure 9 introduces various approaches to suppress the leakage current from the parasitic channel without the formation of a GP region [14,25]. A full bottom dielectric (FBD) can eliminate the parasitic channel perfectly (Figure 9a). However, fabrication of the FBD inevitably requires a starting wafer composed of silicon-on-insulator (SOI) which is vulnerable to self-heating as well as expensive wafer cost. In this context, a steep-retrograde (SSR) region which contains a deep and heavily doped layer is preferred. However, forming an abrupt doping profile for the SSR is impossible using ion implantation and spike annealing. Even though epitaxial growth can be utilized, at least one more epitaxial growth step is required compared to the DUT case. Figure 9d shows the simulated electrical characteristics of the FBD, SSR, and DUT structures, respectively. Even though the DUT layer has a simpler fabrication process than the others, device characteristics in terms of *V*_TH_, *SS*, and *I*_ON_ are not remarkable. Exact device parameters are summarized in Table 3.

## 4. Conclusions

For better process simplification, the fabrication process for nanosheet FETs was newly suggested based on 3-D simulation. The doped ultra-thin (DUT) layer can be epitaxially grown in-situ on the starting wafer. Conventional ground plane (GP) doping implantation as well as annealing process can be excluded while forming the DUT layer. The thickness of the DUT layer has been optimized to suppress parasitic channel leakage, punch-through, and band-to-band tunneling (BTBT). The NS FET with the DUT layer showed comparable performance, but has a simpler fabrication process compared with other NS FETs, including full bottom dielectric (FBD) or steep-retrograde region (SSR).

## Figures and Tables

**Table 1:** Dimensions and parameters used for the TCAD simulations.

Parameter	Value
Gate Length, *L*_G_	12 nm
Nanosheet Width, *W*_NS_	45 nm
Inner Spacer Thickness, *T*_SPACER_	3 nm
Nanosheet-to-Nanosheet Vertical Space, *V*_SPC_	10 nm
Nanosheet Thickness, *T*_NS_	5 nm
Doped Ultra-Thin (DUT) Layer Thickness, *T*_DUT_	5–100 nm
Doping Concentration of DUT Layer (*N*_DUT_)	10^19^ cm^−3^
Inter Layer SiO_2_ Thickness, *T*_IL_	0.5 nm
High-*k* Gate Dielectric Thickness, *T*_HK_	1.28 nm
Contacted Poly-Si Pitch (CPP)	44 nm

**Table 2:** Comparison of DIBL and *SS* characteristics with various devices.

	Planar FET [19]	FinFET [24]	NS FET [This Work]
DIBL (mV/V) (*V*_D_ = 50 mV and 0.7 V)	208	89	35
*SS* (mV/dec)	-	72	69

**Table 3:** Comparison of NS FETs based on FBD, SSR, and DUT structures.

	FBD	SSR	DUT
*V*_TH_ (0.7 V/50 mV) (mV)	212/229	212/233	216/239
*SS* (mV/dec)	69	69	69
*I*_ON_ (mA) at *V*_G_ = 0.7 V, *V*_D_ = 0.7 V	0.117	0.117	0.102
*I*_OFF_ (pA) at *V*_G_ = 0 V, *V*_D_ = 0.7 V	71.5	71.0	65.3
DIBL (mV/V) (*V*_D_ = 50 mV and 0.7 V)	32	32	35

## Data Availability

Not applicable.

## References

[1] Chang L., Choi Y.-K., Ha D., Ranade P., Xiong S., Bokor J., Hu C., King T.-J. (2003). Extremely scaled silicon nano-CMOS device. Proc. IEEE.

[2] Razavieh A., Zeitzoff P., Nowak E.J. (2019). Challenges and Limitations of CMOS Scaling for FinFET and beyond Architectures. IEEE Trans. Nanotechnol..

[3] Hisamoto D., Lee W., Kedzierski J., Takeuchi H., Asano K., Kuo C., Anderson E., King T.J., Bokor J., Hu C. (2000). FinFET—A self-aligned double-gate MOSFET scalable to 20 nm. IEEE Trans. Electron Devices.

[4] Lee S.Y., Kim S.M., Yoon E.J., Oh C.W., Chung I., Park D., Kim K. (2004). A novel multi-bridge-channel MOSFET (MBCFET): Fabrication technologies and characteristics. IEEE Trans. Nanotechnol..

[5] Bae G., Bae D.-I., Kang M., Hwang S.M., Kim S.S., Seo B., Kwon T.Y., Lee T.J., Moon C., Choi Y.M. 3nm GAA Technology featuring Multi-Bridge-Channel FET for Low Power and High Performance Applications. Proceedings of the 2018 IEEE International Electron Devices Meeting (IEDM).

[6] Loubet N., Hook T., Montanini P., Yeung C., Kanakasabapathy S., Guillom M., Yamashita T., Zhang J., Miao X., Wang J. Stacked nanosheet gate-all-around transistor to enable scaling beyond FinFET. Proceedings of the 2017 IEEE Symposium on VLSI Technology.

[7] Witters L., Veloso A., Ferain I., Demand M., Collaert N., Son N.J., Adelmann C., Meersschaut J., Vos R., Rohr E. Multiple-Vt FinFET devices through La_2_O_3_ dielectric capping. Proceedings of the IEEE International SOI Conference.

[8] Li J., Li Y., Zhou N., Xiong W., Wang G. (2020). Study of Silicon Nitride Inner Spacer Formation in Process of Gate-all-around Nano-transistors. Nanomaterials.

[9] Lee K.-S., Park J.-Y. (2021). Inner Spacer Engineering to Improve Mechanical Stability in Channel-Release Process of Nanosheet FETs. Electronics.

[10] Ritzenthaler R., Mertens H., De Keersgieter A., Mitard J., Mocuta D., Horiguchi N. Isolation of nanowires made on bulk wafers by ground plane doping. Proceedings of the 2017 47th European Solid-State Device Research Conference (ESSDERC).

[11] Choi Y., Lee K., Kim K.Y., Kim S., Lee J., Lee R., Kim H.-M., Song Y.S., Kim S., Lee J.-H. (2020). Simulation of the effect of parasitic channel height on characteristics of stacked gate-all-around nanosheet FET. Solid State Electron..

[12] Hong J.M., Park J.W., Lee J.W., Ham J.H., Park K.R., Jeon J.W. (2019). Alpha Particle Effect on Multi-Nanosheet Tunneling Field-Effect Transistor at 3-nm Technology Node. Micromachines.

[13] Seon Y., Chang J., Yoo C., Jeon J. (2021). Device and Circuit Exploration of Multi-Nanosheet Transistor for Sub-3 nm Technology Node. Electronics.

[14] Jegadheesan V., Sivasankaran K., Konar A. (2020). Optimized Substrate for Improved Performance of Stacked Nanosheet Field-Effect Transistor. IEEE Trans. Electron Devices.

[15] Ang K.-W., Barnett J., Loh W.-Y., Huang J., Min B.-G., Hung P.Y., Ok I., Yum J.H., Bersuker G., Rodgers M. 300 mm FinFET results utilizing conformal, damage free, ultra shallow junctions (Xj∼5 nm) formed with molecular monolayer doping technique. Proceedings of the 2011 IEEE International Electron Devices Meeting (IEDM).

[16] Chen W.C., Lin H.C., Chang Y.C., Lin C.D., Huang T.Y. (2010). In Situ Doped Source/Drain for Performance Enhancement of Double-Gated Poly-Si Nanowire Transistors. IEEE Trans. Electron Devices.

[17] Ancona M.G., Iafrate G.J. (1989). Quantum correction to the equation of state of an electron gas in a semiconductor. Phys. Rev. B..

[18] Huang H.S., Wang W.L., Wang M.C., Chao Y.H., Wang S.J., Chen S.Y. (2018). I-V model of nano nMOSFETs incorporating drift and diffusion current. Vacuum.

[19] Chao S.-Y., Huang H.-S., Huang P.-R., Lin C.-Y., Wang M.-C. (2022). Channel Mobility Model of Nano-Node MOSFETs Incorporating Drain-and-Gate Electric Fields. Crystals.

[20] Woltjer R., Tiemeijer L., Klaassen D. (2007). An industrial view on compact modeling. Solid-State Electron..

[21] Slotboom J.W., De Graaff H.C. (1977). Bandgap Narrowing in Silicon Bipolar Transistors. IEEE Trans. Electron Devices.

[22] (2016). Sentaurus Device User Guide, Version L-2016.03.

[23] Yoon J.-S., Jeong J.S., Lee S.H., Lee J.J., Lee S.G., Lim J.W., Baek R.H. (2022). DC Performance Variations by Grain Boundary in Source/Drain Epitaxy of sub-3-nm Nanosheet Field-Effect Transistors. IEEE Access.

[24] Xie R., Montanini P., Akarvardar K., Tripathi N., Haran1 B., Johnson S., Hook T., Hamieh B., Corliss D., Wang J. A 7 nm FinFET technology featuring EUV patterning and dual strained high mobility channels. Proceedings of the 2016 IEEE International Electron Devices Meeting (IEDM).

[25] Zhang J., Frougier J., Greene A. Full Bottom Dielectric Isolation to Enable Stacked Nanosheet Transistor for Low Power and High Performance Applications. Proceedings of the IEEE 2019 IEEE International Electron Devices Meeting (IEDM).

